# Nurturing Societal Values in and Through Health Innovations: Comment on "What Health System Challenges Should Responsible Innovation in Health Address?"

**DOI:** 10.15171/ijhpm.2019.57

**Published:** 2019-07-07

**Authors:** Payam Abrishami, Sjoerd Repping

**Affiliations:** ^1^National Health Care Institute, Diemen, The Netherlands.; ^2^Amsterdam University Medical Centre, Amsterdam, The Netherlands.

**Keywords:** Innovation Policy, Stakeholder Participation, Social Entrepreneurship, Health Technology Assessment, Responsible Innovation

## Abstract

Aligning innovation processes in healthcare with health system demands is a societal objective, not always achieved. In line with earlier contributions, Lehoux et al outline priorities for research, public communication, and policy action to achieve this objective. We endorse setting these priorities, while also highlighting a ‘commitment gap’ in collectively addressing system-level challenges. To acknowledge that stakeholders engaged in dialogue with one another are addressing the commitment gap is not a small step but a giant leap towards realising a socially responsible innovation agenda. Translating system-level demand signals into innovation opportunities is, therefore, the task-cum-art of all stakeholders, one that often prompts them to innovate how they deal with innovations.

Advances in healthcare technology over the last 50 years have contributed to improving life expectancy, reducing patients morbidities, and offering professionals opportunities to improve the quality and efficiency of healthcare.^1^ The influx of innovations into publicly-funded healthcare systems continues and horizons are constantly being pushed. ‘Innovation’ carries a strong connotation of ‘novelty’ and recency.^2^ Although this conceptualisation has dominated present-day thinking about innovation in healthcare, it would be naive to maintain that changing to whatever is ‘new’ is inherently good. True innovation must be a change for the *better*.

## Is “New” Invariably “Better”?

While an innovation’s recency seems straightforward, its betterment is often not.

First, diverse stakeholders involved in the development, adoption, spread, and regulation of new technology hold diverse visions on an innovation’s value, ranging from achieving market competition and entrepreneurship, to promoting population health, equitable access, and universal coverage.^3-5^ These visions often collide in theory and practice. For instance, a policy aimed at ‘pushing’ entrepreneurial start-ups to create business value, like in the case of medical and wellness apps, might result in innovations that are suboptimal in creating aggregate social value.^3,6,7^

Second, many innovative surgical devices, treatment strategies, imaging equipment, apps, etc are adopted and used in decentralised arrangements while their clinical benefits and value for the costs they incur to the health system remain largely understudied.^8-10^ Existing regulations for market entry and acquisition practices of in-hospital and digital technologies do not often require substantial evidence of (cost)effectiveness up-front.

Third, implementation is ‘open-ended’ as in the case of predictive AI algorithms, hybrid operating rooms, gene editing, etc. The route from an innovation’s ‘black boxed’ promises to actual value is convoluted and conditional to, amongst others, an appropriate infrastructure, training, users’ experience, safety and privacy protocols, liability, business model, and maintenance.^2,3^

Fourth, dissemination of new diagnostic and therapeutic technologies can contribute, in a self-perpetuating fashion, to unrealistic expectations, inflated patient demands, increased health anxiety, over-treatment, and medicalisation. Hence, ethical suitability in terms of how an innovation shapes the ideals of social service delivery, definitions of human disease, and patterns of resource allocation in healthcare are also often unclear and under-examined.^11,12^

Fifth, medical innovations raise concerns about the affordability of both well-developed and developing healthcare systems. They drive expenditure growth, being responsible on average for half the annual increase in healthcare costs.^13,14^ Spreading at an inelastic, high unit price (eg, surgical robots), growing use (CT/MRI scans), or expanding indications (heart implants) can explain expenditure growth.^4,15^ Moreover, new technologies may attract many resources within and beyond adopting organisations, thereby eliminating the provision of ‘less lucrative’ services (eg, counselling).^11,16^ Concerns about affordability and access can jeopardise citizens’ support for maintaining a publicly-funded healthcare system, hence shaking the pillars of social solidarity.

Just as healthcare innovations may be part of the ‘solution’ by promising some benefits, they may also be part of the ‘problem’ by causing – sometimes far-reaching – consequences to the healthcare system, particularly if the investment is high, ethical issues are at stake, or the added value for patient is limited.^2^ An innovation does not automatically lead to betterment when its value to society at large is at stake.

## Begin With the End in Mind!

The article by Lehoux et al is an important contribution to dealing with the problem of medical technology, ie, achieving betterment from innovations.^6^ Drawing on the principles of responsible research and innovation, the article emphasises the issue of aligning healthcare innovations with public healthcare system objectives.

The conceptual core of the article is almost self-evident: insofar as new technologies are provided within public healthcare systems, they need to contribute to population health, quality of care, access, and financial sustainability. However, Lehoux et al wonder why system-level challenges have so far not been adequately addressed. We find this much less surprising given the explanations the article itself provides. The bottom-line is this: ‘current incentives, practices of technology introduction, and regulation mechanisms are not well supporting or rewarding the public goods that health systems need.’^6^

The article contributes to the theory and practice of responsible innovation in healthcare in the following respects: signalling obstacles, describing priorities, and recommending policy proposals. The obstacles include (*i*) a research gap partly because literature is scattered across different scientific disciplines, geographies, patient groups and services, (*ii*) a communication gap in articulating system-level priorities explicitly, and (*iii*) an action gap in setting societal challenges at the outset when developing innovation trajectories in healthcare. To this, we add (*iv*) an overarching *commitment* gap in collectively filling the above-mentioned gaps. The article attempts to fill the research gap by providing a comprehensive scoping review of system-level challenges in different health systems across the Human Development Index spectrum. Using the ‘dynamic health system’ analytical framework, the authors explicitly articulate the ‘demand’ (priorities) for innovation from a public health system perspective. In addition, a range of policy initiatives at different stages of the innovation process are recommended to address the action gap.^6^

## A “Giant Leap” Forward

We endorse the priorities for research, public communication, and policy action provided by Lehoux et al and believe these are essential building blocks for making an innovation *agenda*. However, commitment matters. The authors emphasise that ‘translating system-level demand signals into innovation opportunities’ is the task of policy-makers. We believe this is the task-cum-art^[1]^ of *all* stakeholders including technology developers, users/patients, assessors/payers, and policy-makers. Once health system challenges are placed centre stage throughout the innovation process, a *shared* agenda can be formed in accordance with responsible innovation in healthcare. Stakeholders can then engage in deliberation as to how to align on and *co-create* social value from innovations.^3^ Co-creating value may not prove easy, given the divergent interests and dispersed tasks; acknowledging it as a responsibility of us all, however, is key to addressing the commitment gap.^3^ This is not a small step but a giant leap towards realising a socially responsible innovation agenda.

Rather than regarding health system priorities as ‘hurdles’ to innovation or ‘valleys of death,’ investors and technology developers can embrace them. A distinctive business logic involves: catering for system-level demands (eg, access) to develop readily-scalable solutions rather than niche marketing. This means prioritising service innovation (eg, digital patient-referral or waiting-list solutions) and making intangibles (eg, patents, algorithms, return on investment models) responsive to public health priority problems; in other words shifting from ‘fancy’ to ‘frugal’ and ‘less sexy’ innovations.^3,6,17,18^ As a result, social entrepreneurial strategies can be devised that enhance rather than limit commercial opportunities. Along with a duty at the outset to respond to system-level challenges comes the advantage of getting the innovation publicly-procured and scaled up within a healthcare system.

Setting system-level challenges at the outset also has important capacity-building implications for health technology assessment (HTA).^19^ It helps enrich the role, methodology, and future direction of HTA as the knowledge tool situated at the intersection of research and innovation policy. This opens up new foci in HTA, characterised by attentiveness to real-world practices, involvement in the early stages of innovations, and incorporation of insights from ‘constructive technology assessment’ and ‘value-based healthcare.’^3,20^ This is in accordance with the need for an evaluative knowledge within the innovation ecosystem that is adaptive (no one-size-fits-all), reflexive, and anticipatory.^6,21^

Public communication on health system objectives enhances public awareness of these objectives and strengthens a critical scrutiny of (supply-induced) demands for an innovation in the face of seductive advertisements.^22^ It is the appreciation of health system challenges that can prompt users/adopters of an innovation to become more self-reflexive regarding their own expectations of innovation and system-level consequences of their decisions. In addition, cultural change agents and pedagogic communication experts can, similar to health promotion endeavours, contribute to promoting the mind-set of appropriate use of innovations in clinical practice and medical curricula.

Finally, a “translation” of system-level demands into innovation priorities by policy-makers renders a complex mix of governance and social-marketing initiatives.^6,23^ This includes the following: streamlining existing local policies by partnering the organisations involved in innovation policy with those of public health policy, facilitating interaction between stakeholders on the innovation’s push-side (entrepreneurship) and pull-side (public health), upscaling innovations within conditional access schemes while also harnessing big-data potentials for an outcome-based financing, valorising and financially rewarding social value-driven intangibles, and prioritising public debate on the societal desirability and appropriate use of new treatments. Policy-makers and public authorities will accordingly need to adjust their modus operandi from performing as ad hoc, reactive rule-setters to all-round, proactive *moderators* of innovation practices towards achieving socially desirable ends.^3^ To do so, policy-makers may need to innovate how they communicate and regulate innovations.

## Ethical issues

Not applicable.

## Competing interests

Authors declare that they have no competing interests.

## Authors’ contributions

Both authors contributed to the concept and writing of the article. PA drafted the manuscript and SR revised and reviewed it.

## Authors’ affiliations

^1^National Health Care Institute, Diemen, The Netherlands. ^2^Amsterdam University Medical Centre, Amsterdam, The Netherlands.

## Endnote


[1] The task-cum-art of translating health system demands into innovation opportunities is not a mere technical task, but one requiring compassion, commitment, courage, and creativity.
